# Detect it so you can treat it: A case series and proposed checklist to detect neurotoxicity in checkpoint therapy

**DOI:** 10.1016/j.ensci.2021.100324

**Published:** 2021-02-01

**Authors:** Saskia Bolz, Thivyah Ramakrishnan, Michael Fleischer, Elisabeth Livingstone, Benjamin Stolte, Andreas Thimm, Kathrin Kizina, Selma Ugurel, Christoph Kleinschnitz, Martin Glas, Lisa Zimmer, Tim Hagenacker

**Affiliations:** a Department of Neurology, Essen University Hospital, Hufelandstrasse 55, 45147 Essen, Germany; b Department of Dermatology, Essen University Hospital, Hufelandstrasse 55, 45147 Essen, Germany; c Division of Clinical Neurooncology, Department of Neurology, Essen University Hospital, Hufelandstrasse 55, 45147 Essen, Germany; d German Cancer Consortium (DKTK), Heidelberg, Germany

**Keywords:** Melanoma, Checkpoint inhibitor, Nivolumab, Ipilimumab, Neurotoxicity, Guide, AIDP, acute inflammatory demyelinating polyneuropathy, anti-CTLA-4, anti-cytotoxic T-lymphocyte-associated protein 4, anti-PD-1, anti-programmed cell death protein 1, CIDP, chronic inflammatory demyelinating polyneuropathy, CNS, central nervous system, CSF, cerebrospinal fluid, ICI, immune checkpoint inhibitor, irAE, immune-related adverse events, i.v, intravenous, IVIG, intravenous immunoglobulin, MG, Myasthenia Gravis, MRI, magnetic resonance imaging, PD-L1, programmed cell death protein 1 ligand

## Abstract

**Background:**

Checkpoint inhibitors show impressive and durable responses in various cancer types and provide new avenues for cancer immunotherapy. However, these drugs have a variety of adverse events. Common autoimmune-related adverse effects include fatigue, hepatitis, skin rash, endocrine deficiencies, and colitis. Neurotoxicity has been reported, but its incidence and course remain unclear.

**Methods:**

To illustrate the broad spectrum of neurotoxicity, we exemplarily report the neurological adverse events of five patients with melanoma and one patient with differentiated thyroid cancer who received checkpoint inhibitors at Essen University Hospital (Essen, Germany).

**Results:**

After treatment with ipilimumab, nivolumab or pembrolizumab, neurotoxic effects included hypophysitis-associated neck pain and headache, Guillain-Barré syndrome, transverse myelitis, acute brachial plexus neuritis, and ocular myasthenia gravis.

**Conclusions:**

Checkpoint inhibitor therapy remains a success story; however, neurological immune-related adverse events may cause severe life-threatening conditions. We propose a guide for the early detection of neurological adverse events during routine clinical treatment to prevent more severe courses of checkpoint inhibitor-induced neurotoxicity.

## Highlights

• We present neurological immune-related adverse events under checkpoint-inhibitors to underline the spectrums of manifestations.
• Neurological immune-related adverse events may cause severe life-threatening conditions.
• Practitioners should be aware of red flags symptoms to detect neurological immune-related adverse events

## 1 Background

Immune checkpoint inhibitors (ICIs) in cancer therapy have significant clinical benefits and result in higher survival rates in patients. Among the approved ICIs, drugs targeting anti-cytotoxic T-lymphocyte-associated protein 4 (anti-CTLA-4) and anti-programmed cell death protein 1 (anti-PD-1) are widely implemented in routine clinical treatment. The ICIs suppress inhibitory pathways that block effective anti-tumour T-cell responses. The targeted ‘immune checkpoints’ control disproportionate excessive immune activation and tumour cells seem to use them as a vehicle to evade the immune system [1]. Ipilimumab was one of the first anti-CTLA-4 ICI to significantly improve disease outcomes in patients with advanced melanoma [2].

Nivolumab and pembrolizumab block the PD-1 receptor and thus suppress negative regulatory pathways in T-cell responses. Programmed cell death protein 1 and its ligand (PD-L1) inhibit T-cell activation and affect cell death by diminishing cell growth factors and survival signals [[3], [4], [5]]. Genetic polymorphisms in the PD-1 locus in humans increase the risk of various autoimmune diseases [6,7]. Immune-related adverse events (irAE) in checkpoint therapy may result from impeded self-tolerance from the loss of T-cell inhibition as a predictable side effect [8,9]. ‘Removing the brakes’ of the immune systems has resulted in various irAEs. The most frequent are colitis, hepatitis, skin rashes or other dermatological complications, interstitial pneumonitis, and endocrine deficiencies, such as thyroiditis or hypophysitis. In general, irAEs seem to occur at a higher rate with CTLA-4 blockade than with anti-PD-1 therapy and at a greater degree of severity when combining CTLA-4 and PD-1 blockade [10,11]. Neurological irAEs are less common, with an incidence of 0.9%–14% [12,13], and can result in severe or fatal complications [[14], [15], [16]].

To illustrate the broad spectrum of neurotoxicity after ICI therapy, we present a case series of six cancer patients who experienced neurological irAEs. We also propose a flowchart to detect neurologic irAEs as early as possible during routine clinical treatment.

## 2 Methods

All patients treated with ICIs at the Departments of Dermatology, Endocrinology, and Neurology at Essen University Hospital (Essen, Germany) from 2015 to 2019 were retrospectively reviewed. Five patients with malignant melanoma and one patient with thyroid cancer were identified with exemplary neurological irAEs. They were continuously monitored and evaluated during and after ICI treatment. Cases #1 and #5 were enrolled in ongoing double-blind clinical trials; therefore, the actual ICI used was unknown. Data were extracted by clinical examination protocols and chart review. According to the local regulation (Essen University ethics committee), ethics committee approval was not required to perform retrospective data analysis.

## 3 Results

### 3.1 Case #1: excruciating neck pain and headache caused by hypophysitis

A 40-year-old woman was enrolled in a double-blind clinical trial comparing adjuvant ipilimumab and nivolumab versus nivolumab monotherapy in patients with metastatic melanoma. After 2 months, the patient complained of new and intense neck pain, holocephalic headache and signs of fatigue. The neurological examination was unremarkable. Her medical history was negative for signs of a primary headache disorder. Cervical and cerebral magnetic resonance imaging (MRI) scans showed no significant changes, laboratory testing including CK levels (to exclude myositis) and CSF parameters were normal. Laboratory work up revealed autoimmune hypophysitis with adrenal insufficiency. Hormone replacement therapy was immediately initiated, including an intravenous (i.v.) prednisolone bolus (100 mg), followed by hydrocortisone substitution (30 mg daily) and slow tapering to 15 mg daily (Table 1). The neurological symptoms rapidly and completely resolved. Ten days later, blood analysis revealed thyroid dysfunction. The pituitary dysfunction continued for several months; therefore, chronic hypophysitis was diagnosed. However, neurological symptoms did not recur.

**Table 1:** Patients' characteristics and the clinical findings of neurological irAEs associated with ICI therapy.

	Case 1	Case 2	Case 3	Case 4	Case 5	Case 6
Age	40	72	55	51	27	56
Sex	Female	Male	Male	Female	Female	Male
Tumour	Metastatic melanoma (MM)	MM	MM	MM	MM	Differentiated thyroid cancer
Checkpoint	Nivolumab + Ipilimumab vs. Nivolumab	Nivolumab + Ipilimumab vs.	Ipilimumab	Ipilimumab +	Nivolumab + Ipilimumab vs. Nivolumab	Pembrolizumab
		Nivolumab,				
Inhibitor				Nivolumab,followed by Nivolumab monotherapy		
		Ipilimumab +				
		Nivolumab				
Time of initiation to symptoms	2 months	1,5 months	2 months	11 months	8 months	0,75 months
Clinical features	Frontal localized, pressing headache, fatigue	Bilateral facial nerve paralysis, reduced tendon reflexes, positive Romberg sign, mild dysmetria of upper extremities	Hypoesthesia lower extremities, paresis, exaggerated tendon reflexes, clonus	Pain and weakness of left arm, reduced tendon reflexes	Periaxillary pain and weakness of left hand, hypoesthesia reduced reflexes	Diplopia, bilateral ptosis
Diagnosis	Neck pain and headache attributed to hypophysitis	Guillain-Barré syndrome	Transverse Myelitis	Brachial plexus neuritis	Brachial plexus neuritis	Ocular myasthenia gravis
Diagnostics	Cortisol, ACTH, TSH, fT3, fT4, IGF-1, LH, FHS, estradiol; cranial MRI	CSF with pleocytosis, protein level of 98 mg/dl, demyelinating polyneuropathy	CSF with mild pleocytosis, contrast enhancement C1 and C3-5 in spinal MRI	CSF with mild pleocytosis	denied	Myasthenia gravis-associated antibodies negative, decrement not detectable
Treatment	100 mg prednisolone i.v. bolus + hydrocortisone substitution	1 g methylprednisolone i.v. for 3d + IVIG (0,4 g/kg/d for 5 d)	1 g methylprednisolone i.v. for 5d + taper over 4 weeks	1 g methylprednisolone i.v. for 3d	100 mg prednisolone p.o. + taper over 8 weeks	prednisolone 1 mg/kg/d p.o. + taper over 6 weeks + pyridostigmin
Oncological outcome	Progression of metastases	Progression of metastases	Complete remission	Stable tumour and pulmonary metastasis	Stable	Stable
Neurological outcome	Complete	Partially resolved with sequelae	Excellent, mild residual Hypoesthesia, vivid reflexes	Complete	Complete	Excellent, mild residual ptosis on one side
Follow-up period	16 months	1 month	4 years	15 months	12 months	5 months

### 3.2 Case #2: bilateral facial nerve palsy in a case of Guillain-Barré syndrome

**Fig. 1:**
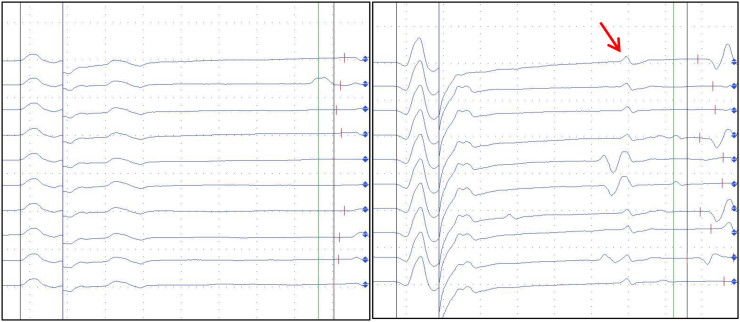
Electroneurography of several nerves show F-wave latencies and A-waves in the left peroneal nerve (left) and tibial nerve (right).

A 72-year-old man with melanoma was treated within the same adjuvant trial as in Case #1. Owing to pulmonary and cerebral disease progression, treatment within the trial was stopped. The patient was switched to combination immunotherapy with ipilimumab (3 mg/kg) and nivolumab (1 mg/kg). After the third infusion of combination therapy, he presented with slurred speech and incomplete eye lid closure due to bilateral facial nerve palsy. Five to 6 weeks before symptom onset, he experienced a few days of fever, headache, adynamia and loss of appetite. An examination revealed mild deep tendon reflexes and loss of the Achilles' tendon reflex, positive Romberg's sign and mild dysmetria of the upper extremities. Electrophysiological studies revealed prolonged F-wave latencies in several nerves, A-waves, reduced nerve conduction velocity and prolonged distal motor latency delay consistent with demyelinating polyneuropathy (Fig. 1). The cerebrospinal fluid (CSF) had pleocytosis (20 leukocytes/μL) and an elevated protein level (98 mg/dl). Inflammation and meningeal carcinomatosis were absent in cranial MRI studies.

For 3 consecutive days, methylprednisolone (1 g i.v.) was administered daily. His symptoms rapidly improved and therapy was continued with intravenous immunoglobulin for 5 days (IVIG 0,4 g/kg/day).

### 3.3 Case #3: transverse myelitis

**Fig. 2:**
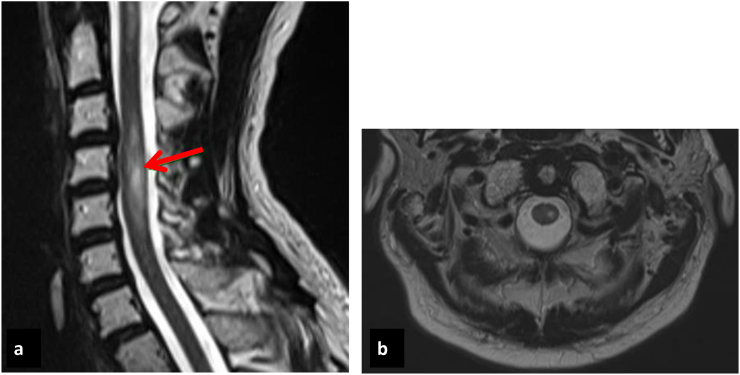
Axial (a) and sagittal (b) contrast magnetic resonance image findings of C1 and C3-5 using the T-2 weighted sequence.

In 2015, a 55-year-old man was diagnosed with malignant melanoma with cervical and pulmonary metastasis. At that time, he was treated with ipilimumab. After the second infusion and 2 months after initiating ICI therapy, the patient complained of sudden loss of sensibility in his hands, gluteal region and legs, primarily on the left side, and weakness of his left hand. Neurological examination showed exaggerated deep tendon reflexes, clonus of the lower extremity and weakness in his left lower and in both upper extremities. He presented hypoesthesia in dermatomes C5–T1 on the left side and dermatomes L3–S2 on the right side, and from dermatome L2 downwards along the left side, pallanaesthesia of the lower extremities and ataxia. An MRI image of the spine revealed gadolinium enhancement at the C1 and C3-5 spinal cord levels, MRI of the brain was normal. He was diagnosed with acute transverse myelitis (Fig. 2). The CSF showed 8 leukocytes/μl and an elevated protein level (72 mg/dL). Extensive microbacterial and virological studies were negative. Tumour cells were also undetectable. Immunosuppressive therapy with methylprednisolone (1 g i.v.) was administered daily for 5 consecutive days. Four days later, symptoms improved. Corticosteroids were tapered during the following 4 weeks. The follow-up examinations revealed residual hypoesthesia and paraesthesia, and slightly reduced strength of the left hand and vivid reflexes. In terms of tumour growth, the patient had a complete response to ipilimumab and remains without a relapse 4 years later.

### 3.4 Cases #4 and #5: brachial plexus neuritis

Two patients with malignant melanoma were neurologically asymptomatic when nivolumab and ipilimumab, respectively, were administered. The first patient (Case #4), a 51-year-old woman, developed autoimmune hepatitis, autoimmune colitis and thromboembolic incidents after receiving a combined therapy with nivolumab (1 mg/kg) and ipilimumab (3 mg/kg). After symptoms resolved, the therapy regimen continued with biweekly nivolumab monotherapy (3 mg/kg). After 11 months following the 20th infusion of nivolumab (3 mg/kg), she experienced massive pain in the left shoulder and neck area, which spread to the left arm. She presented to the neurology department after developing severe proximal and moderate distal weakness of the extremities. Pain was rated as 8–9 of 10 on a numeric rating scale. The left brachioradialis reflex was reduced. Electrophysiological studies conducted 5 days after symptom onset revealed no signs of chronic nerve damage in keeping with acute idiopathic plexus neuritis. The CSF had mild pleocytosis (5 leukocytes/μl) with a normal protein level. Viral and bacterial pathogens and tumour cells were undetectable. Paresis rapidly responded within 3 days of intravenous methylprednisolone (1 g/day). No symptoms recurred with continued ICI therapy. The second patient (Case #5), a 27-year-old woman, received ipilimumab and nivolumab simultaneously or nivolumab as monotherapy in a randomized double-blind clinical trial. Eight months after beginning treatment, she complained of having pain in the left armpit and periaxillary area for more than 1 week when numbness, paraesthesia of the fourth and fifth finger along with mildly reduced strength in flexion and spreading of fingers of the left hand occurred. The left brachioradialis reflex was reduced; therefore, brachial plexus neuritis was diagnosed. The patient refused lumbar puncture and intravenous corticoids. However, the patient responded rapidly to oral steroids (100 mg prednisolone for 3 days and tapering over 8 weeks) and showed normal function of the left extremity within several days of treatment. Both patients continued ICI treatment 3 weeks after the onset of complaints. The neuritis did not recur.

### 3.5 Case #6: ocular myasthenia gravis

A 56-year-old man was diagnosed with thyroid cancer and pulmonary metastases. He was treated with pembrolizumab off-label after tumour progression, after previously undergoing radioiodine treatment, and levatinib and sorafenib therapy. Within 36 h following the second pembrolizumab infusion and 3.5 weeks after initiating treatment, the patient developed diplopia, which worsened within 1 day. Ten days later, he had right sided ptosis, which caused near-complete eye closure. Over several days, he also developed incomplete ptosis on the left side. Dysarthria, dysphagia, or weakness of the extremities was absent. When he presented to our department, his Quantitative Myasthenia Gravis score was 10 points (of 39 points), which indicated ICI-induced myasthenia gravis (MG). Autoantibodies against neuromuscular junction proteins were negative, CK was not elevated. Repetitive nerve stimulation of the left facial nerve showed no decrement and cerebral MRI scans were not indicative. An oral pyridostigmin test (60 mg) was positive with immediate improvement of ptosis. His symptoms rapidly improved with prednisone (1 mg/kg/day), followed by slow tapering and symptomatic cholinesterase inhibitor therapy (pyridostigminbromide, 180 mg/day). During the following 6 weeks, no indication of recurrence or generalization of MG occurred. Therefore, pembrolizumab therapy was continued.

## 4 Discussion

In this case series of six cancer patients who experienced neurologic irAEs, we illustrated the broad spectrum of neurotoxicity after ICI therapy and the treatment response for these adverse effects. Due to the increasing use in treatment of ICI in melanoma, 5 of 6 patients were suffering from this type of cancer. Based on these findings, we propose a flowchart to detect neurological irAEs as early as possible during routine clinical treatment. Neurological complications potentially related to ICI therapy vary from myositis [17,18] and MG [[19], [20], [21]]to inflammatory demyelinating polyneuropathies [[22], [23], [24], [25]] to granulomatous inflammation of the CNS [21], multifocal CNS demyelination [26], meningitis [22], myelitis [19] and encephalopathy [13]. In particular, when ICI is implemented by non-neurological professionals, neurotoxicity can be difficult to diagnose and may therefore be underreported. The data on incidence of irAE is very limited [18].

Hypophysitis is a common irAE with ICI therapy, and its overall incidence is up to 9% [27]. However, neurological symptoms are often considered secondary. In Case #1, the misleading symptom of neck pain and cephalgia led to a diagnosis of endocrine dysfunction.

Case #3 illustrates a possible irAE affecting the central nervous system. Transverse myelitis occurred after only two doses of ipilimumab. A similar course of adverse events, which occurred 2 months after initiating ipilimumab therapy, was described in a case of transverse myelitis at spinal cord level T9-10 [25]. When high-dose intravenous steroids were administered, the patient recovered to a certain extent throughout a 2-week period and ICI therapy was withheld. Wilson et al. [28] presented a case of longitudinally extensive transverse myelitis after pembrolizumab treatment in which the patient improved after the intravenous administration of corticosteroids, immunoglobulins and plasma exchange. Further cases have been described [29,30]. Common autoantibody profiles associated with extensive transverse myelitis, such as aquaporin-4 and myelinoligodendrocyte glycoprotein antibodies, were not detected. As in our case, the patient had an excellent neurological outcome during the follow up.

The peripheral nervous system may be particularly vulnerable to immune-mediated toxicity when impaired T-cell reactions occur [31], which is represented by the manifestation of many cases during ICI therapy. Case #2 had a dire irAE because of its potentially fatal course. Similar cases have been reported when ipilimumab, nivolumab, the combination of both, or pembrolizumab was administered. Some of these cases had rapidly ascending symptoms and some cases had a fatal outcome because of multiorgan failure or respiratory insufficiency [22,23,[32], [33], [34]]. With the prodromal phase of fever, a postinfectious aetiology should be taken into account, while the prompt response to steroids favours the irAE hypothesis. Clinicians should consider that, because checkpoint therapies cause irAEs, corticosteroids are a first-line treatment option in treating ICI-induced Guillain-Barré syndrome.

As in Cases #4 and #5, brachial plexus neuritis after PD-1 antibody treatment was reported in one patient after nine infusions of pembrolizumab, and in another patient after nine infusions of nivolumab both with a rebound of symptoms when weaned from corticosteroids and with a rapid response to corticosteroids and a full regaining of function [35]. Compared to idiopathic pathogenesis, ICI-induced plexus neuropathies develop with an acute onset and seem to predominantly affect the lower trunk and muscle atrophy is absent; pain and motor and sensory symptoms respond immediately to high-dose corticosteroids.

Generalized and ocular myasthenic syndromes have been reported with ICI treatment. Fifty-nine percent of patients who developed MG after ICI therapy were positive for antibodies against the neuromuscular junction [38]. Myasthenia gravis can spontaneously develop in CTLA-4 knockout mice [39]. The overexpression of PD-1 potentiates CD8+ T-cell exhaustion; therefore, anti-PD-1 ICI may stimulate the exacerbation of symptoms in patients with pre-existing MG [40]. Myasthenia gravis is a severe condition with an ICI-induced MG-related mortality rate of approximately 30% [38]. In a recent retrospective study authors emphasized the importance of an early diagnosis of neurological irAEs and immediate management with corticosteroids as this was associated with favourable neurological outcomes among these patients. In addition, a majority of 68% of participating patients showed co-occurring non-neurological irAEs, therefore thorough examination of patients with any irAE is crucial in management of ICI therapy [41].

**Fig. 3:**
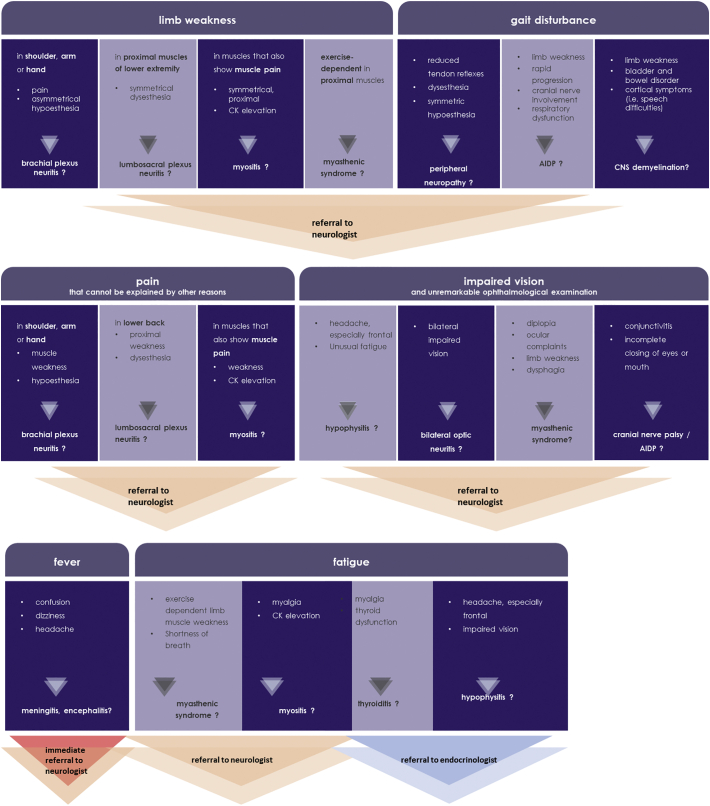
Flow chart to potentially detect and manage neurological irAEs.

The potential severity of neurological irAEs stresses the importance of an attentive screening and examination when treating patients with ICIs. Therefore, we suggest a flowchart—especially for non-neurologists—to detect neurological irAEs as early as possible during clinical routine treatment (Fig. 3).

Given that our findings and flowchart are based entirely on cases series and review of the literature without available prospective data, our results were limited and future prospective studies are needed for validation in the real world.

## 5 Conclusions

All patients in this case series had a significant response to corticosteroid treatment. Despite the severity of some neurologic toxic effects, they are generally treatable and manageable if detected early and treated swiftly with corticosteroids and other immunosuppressive agents, as necessary. ICI treatment was discontinued in the patients with transverse myelitis and Guillain-Barré syndrome. By contrast, for the patients with brachial plexus neuritis and MG, the ICI therapy was continued. Our case series underlines the importance of vigilant and multidisciplinary management of neurological complications with ICI therapy. The proposed checklist with questions for the early detection of neurological irAEs can be useful especially in hospitals where neurologists are not readily available or when neurologists are not usually involved in the assessment of cancer patients during treatment. It should be emphasized that, as suspicion of neuromuscular or central nervous system derangement arises, a neurologist should rapidly be consulted.

## Ethical approval and consent to participate

According to the local regulation (Essen University ethics committee), ethics committee approval was not required to perform retrospective data analysis. All persons gave their informed written consent prior to their inclusion in the study.

## Consent for publication

All patients gave their informed consent for publication of their anonymized data.

## Availability of data and materials

The datasets used and analysed during the current study are available from the corresponding author on reasonable request.

## Funding

None.

## Author contributions

All authors contributed to the study conception and design. Material preparation, data collection and analysis were performed by SB, TR and MF. Data collection and investigation was also performed by BS, AT and KK. The first draft of the manuscript was written by SB and TH. LZ, SU and EL reviewed and edited the manuscript. MG and CK supervised and aided in interpreting he results. All authors commented on previous versions of the manuscript. Also, all authors read and approved the final manuscript.

## Declaration of Competing Interest

SB received travel support from Octapharma (Lachen, Schweiz). EL served as consultant and/or received honoraria from Amgen (Thousand Oaks, CA, USA), Actelion (Allschwil, Switzerland), Roche (Basel, Switzerland), Bristol-Myers Squibb (New York, NY, USA), Merck Sharp & Dohme (Kenilworth, NJ, USA), Novartis (Basel, Switzerland), Janssen (Beerse, Belgium), Medac (Wedel, Germany), and travel support from Amgen, Merck Sharp & Dohme, Bristol-Myers Squibb, Medac, Pierre Fabre (Paris, France), Sunpharma (Mumbai, India) and Novartis. SU declares research support from Bristol-Myers Squibb and Merck Serono (Geneva, Switzerland); speakers and advisory board honoraria from Bristol-Myers Squibb, Merck Sharp & Dohme, Merck Serono, Novartis and Roche, and travel support from Bristol-Myers Squibb, Merck Sharp and Dohme. LZ served as consultant or/and received honoraria from Roche, Bristol-Myers Squibb, Merck Sharp & Dohme, Novartis, Sanofi (Paris, France), and Pierre Fabre, and travel support from Amgen, Merck Sharp & Dohme, Bristol-Myers Squibb, Novartis, Pierre Fabre, and Sanofi. Tim Hagenacker received travel compensation, advisory board and lecture honoraria from CSL Behring (King of Prussia, PA, USA), Biogen and Alexion and is and editorial board member (associate editor) of BMC Neurology. TR, MF, BS, AT, KK, MG and CK have no conflicts of interest.
